# Ligand-induced cold activation of TRPV3

**DOI:** 10.21203/rs.3.rs-5759985/v1

**Published:** 2025-01-21

**Authors:** Guangyu Wang

**Keywords:** ligand exchange, heat capacity, partial unfolding, temperature sensitivity, thermodynamic signature, thermoring stability, protein thermoactivity

## Abstract

Both hot and cold sensation of the homotetrameric thermosensitive transient receptor potential vanilloid 1–4 (TRPV1–4) channels have been predicted by a single Gibbs-Helmholtz equation for a change in molar heat capacity. However, cold activation has not been confirmed for those heat-responsive TRPV1–4 channels. Given the cooperative heat unfolding and non-cooperative cold unfolding behaviors in proteins, two different open states at low and high temperatures should be detected in TRPV1–4 channels. To test this hypothesis, the temperature-dependent quaternary and tertiary structures of oxidized TRPV3 in the presence and absence of the natural cannabinoid tetrahydrocannabivarin (THCV) were characterized along a lipid-dependent minimal gating pathway. Further thermoring analyses showed that gating state-dependent thermostability allowed oxidized TRPV3 to be activated and then inactivated only below 30°C. However, no inactivation would be observed above 30°C once the lipid at the active vanilloid site was released by THCV binding. Therefore, such two temperature-dependent gating pathways of oxidized TRPV3 actually resulted from cold and heat activation. (161 words)

## Introduction

Heat-responsive homotetrameric thermosensitive transient receptor potential vanilloid 1–4 (TRPV1–4) channels have a specific activation threshold and a high temperature sensitivity Q_10_, which is the ratio of rates or open probabilities (P_o_) of an ion channel measured at two temperatures 10°C apart (1). A recent study revealed that reduced or oxidized mouse TRPV3 (mTRPV3) in the absence or presence of the C612-C619 disulfide bond can be activated by heat-induced unfolding of the least-stable K614-N647 in the pore domain (PD) (S5-S6) or the least-stable R416-D519 H-bond in the interface between the pre-S1 domain and voltage sensor-like domain (VSLD) (S1-S4) at the specific melting temperature threshold of 52°C or 40°C, respectively. Furthermore, the specific temperature sensitivity Q_10_ of mTRPV3 is closely related to a change in molar heat capacity (ΔC_p_). For a gating transition from a reduced and closed state to an oxidized and open state, ΔC_p_ is about 8.68 kcal/mol-K. This value decreases to 0.762 kcal/mol-K for an oxidized closed mTRPV3 (2). In this case, based on a single Gibbs-Helmholtz equation, both heat and cold unfolding-induced channel activations of TRPV3 should be observed (3). However, the latter activation may not be directly detected if it occurs at a temperature below the freezing point of water (4).

Previous studies have shown that capsaicin-induced release of a phosphatidylinositol (PI) lipid from an active vanilloid site decreases the heat activation threshold of TRPV1 (5–6). Therefore, when the phosphatidylcholine (PC) lipid at the corresponding vanilloid site is outcompeted by a ligand, TRPV3 is expected to be activated above the freezing point of water. Cold unfolding of globular proteins like ubiquitin and Trp-cage miniprotein, or membrane proteins like PI-free rat TRPV1 (rTRPV1) is non-cooperative, fragmented and locally limited at the protein-water interface. In contrast, global heat unfolding is cooperative (4–9). This suggests that heat and cold unfolding-induced activations of TRPV3 should result in two distinct open states.

To test this hypothesis, cryogenic electron microscopy (cryo-EM) structures of oxidized human TRPV3 (hTRPV3) activated by the natural cannabinoid tetrahydrocannabivarin (THCV) at 4°C were compared with those of heat-activated oxidized mTRPV3 at 42°C (10–11). After identifying two distinct temperature-dependent quaternary structures in the open state, the thermoring structures of THCV-gated oxidized hTRPV3 at 4°C were characterized using a highly-sensitive thermodynamic tool developed and examined in several thermosensitive proteins (2, 6, 9, 12–18). These structures were then compared with those of heat-gated oxidized mTRPV3 at 42°C.

The results showed that the two distinct open states of oxidized hTRPV3 at low and high temperatures had different thermostabilities. This led to inactivation below 30°C and no inactivation above 30°C even in the presence of THCV at the active vanilloid site. These temperature-dependent gating pathways demonstrated that the hot and cold sensing abilities of the thermosensitive TRPV3 channel can be linked and explained by the single Gibbs-Helmholtz equation for a significant ΔC_p_, supporting the heat capacity model.

## Results

### Heat-evoked channel opening at 42 °C rearranges the intersubunit interactions of oxidized mTRPV3

**Figure 1.**
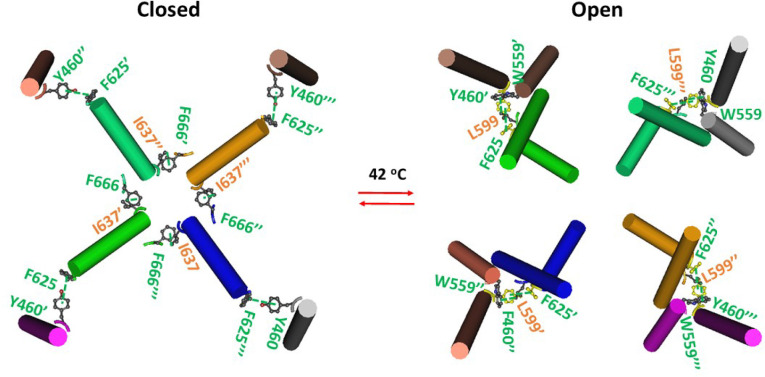
Intersubunit interactions in oxidized mTRPV3 during heat-induced opening. The homo-tetrameric cryo-EM structures of the oxidized mTRPV3 channel in cNW11 nanodiscs in the closed and open states at 42 °C (PDB ID, 7MIN and 7MIO, respectively) were utilized for the model.

In the presence of the PC lipid at the active vanilloid site of the closed homotetrameric mTRPV3 channel with the C612-C619 disulfide bonds in cNW11 nanodiscs at 42 °C, two critical intersubunit interactions were found along the PC-dependent minimal gating pathway from D396 to K705 (2). One was a p-p interaction between F625 from the pore helix of the nth subunit and Y460’ from the VSLD of the (n+1)th subunit; the other was a CH-p interaction between F666 from the PD of the nth subunit and I637’ from the pore helix of the (n+1) subunit. Thus, each PD linked with the neighboring VSLD and PD (Figure 1).

In contrast, in the open state at 42 °C, the swapping F625-Y460’ p interaction remained intact but the swapping I637’-F666 p interaction was replaced by the swapping L599-W559’ p interaction (Figure 1). Hence, the swapping p interaction between the VSLD and the neighboring PD served as an anchor while the swapping p interaction between two neighboring PDs was disrupted for channel opening at high temperature (Figure 1).

### THCV-evoked channel opening at 4 °C rearranges the intersubunit interactions of oxidized hTRPV3 differently

**Figure 2.**
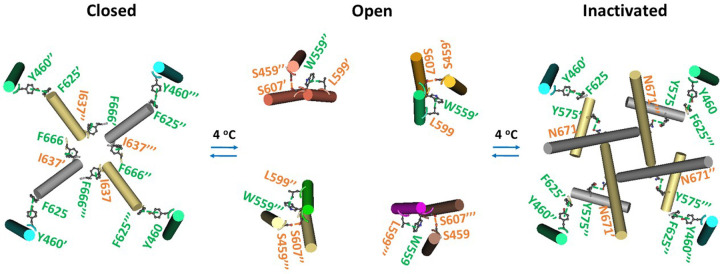
Intersubunit interactions in oxidized hTRPV3 during cold-induced channel gating transitions. The homo-tetrameric cryo-EM structures of the hTRPV3 channel with THCV bound in cNW30 nanodiscs in the closed, open and inactivated states at 4 °C (PDB ID, 8V6K, 8V6L and 8V6M, respectively) were used for the model.

The same intersubunit interactions were also observed in the oxidized closed hTRPV3 channel in cNW30 nanodiscs at 4 °C (Figure 2). When the channel was open with THCV outcompeting the PC lipid from the active vanilloid site, both swapping interactions were replaced by the swapping L599-W559’ p interactions and S607-S459’ H-bond (Figure 2). Notably, an inactivated state also appeared together with the swapping F625-Y460’ and N671-Y575’ p interactions (Figure 2). Therefore, THCV-induced gating transitions of oxidized hTRPV3 at 4 °C were completely different than that of oxidized mTRPV3 at 42 °C despite the same closed state. The next question is if THCV-induced open and inactivated states only exist at a temperature lower than that for heat-evoked channel opening in the same cNW30 nanodiscs.

### Closed oxidized hTRPV3 in cNW30 nanodiscs at 4 °C is unstable in the absence of THCV

**Figure 3:**
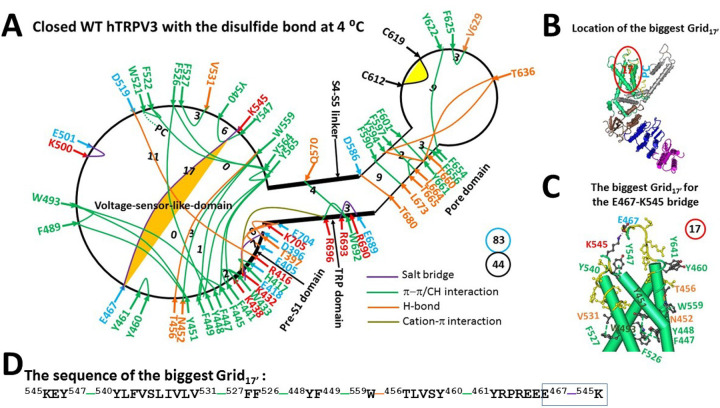
The grid-like noncovalently interacting mesh network along the PC-dependent minimal gating pathway of the oxidized hTRPV3 channel in a closed state at 4 °C. (**A)** The thermoring structure based on cryo-EM data of a single subunit of the oxidized closed hTRPV3 channel in cNW30 at 4 °C (PDB ID, 8V6K). The pore domain, the S4-S5 linker, the TRP domain, and the pre-S1 domain are indicated by black arrows, except the VSLD. Salt bridges, p interactions, and H-bonds between paired amino acid side chains along the PC-dependent minimal gating pathway from D396 to K705 are denoted in purple, green, and orange, respectively. The specific grid sizes necessary to regulate the least-stable noncovalent interactions in the grids are indicated with black numbers. The E467-K545 salt bridge in the biggest Grid_17’_ is emphasized in yellow. The dashed line represents the putative PC against W521. The total grid sizes and the total grid size-controlled noncovalent interactions along the PC-dependent minimal gating pathway are displayed in cyan and black circles, respectively. (**B)** The position of the biggest Grid_17’_. (**C)** The structure of the biggest Grid_17’_ with a 17-residue size to regulate the E467-K545 salt bridge in the VSLD. (**D)** The sequence of the biggest Grid_17’_ to control the E467-K545 salt bridge highlighted in the blue box.

Oxidized closed hTRPV3 in cNW30 nanodiscs at 4 **°**C exhibited similar thermoring structures to oxidized closed mTRPV3 in cNW11 nanodiscs at 42 **°**C (2). However, the distinct PC binding at the active vanilloid site resulted in some differences along the PC-dependent minimal gating pathway from D396 to K705. In cNW11 nanodiscs, PC is sandwiched by W521 and Q695 (2). In contrast, PC only attached to W521 in cNW30 nanodiscs. In addition, the E610-N647-K614-E610 bridges in the PD of closed oxidized mTRPV3 at 42 **°**C were absent in closed oxidized hTRPV3 at 4 **°**C. Finally, the Q570-W692-R696 p interactions were absent in closed oxidized mTRPV3 at 42 **°**C but present in closed oxidized hTRPV3 at 4 **°**C (Figure 3a) (2).

In this case, the total number of grid sizes was 83, much higher than the 64 of closed oxidized mTRPV3 at 42 **°**C in cNW11 nanodiscs. However, the total number of noncovalent interactions was 44, much lower than the 53 of closed oxidized mTRPV3 at 42 **°**C in cNW11 nanodiscs. Thus, the systematic thermal instability (T_i_) was 1.89, higher than the 1.21 of closed oxidized mTRPV3 in cNW11 nanodiscs at 42 **°**C (Table 1).

Notably, the least-stable R416-D519 salt bridge in the biggest Grid_17_ of closed oxidized mTRPV3 in cNW30 nanodiscs at 42 **°**C was accompanied by the H471-Y540-Y547-H471 p interactions (2). In contrast, the same R416-D519 salt bridge of closed oxidized hTRPV3 in cNW30 nanodiscs at 4 **°**C was present along with the least-stable E467-K545 salt bridge controlled by the new biggest Grid_17’_ in the VSLD (Figure 3a–b). It had a 17-residue thermoring from K545 to Y547, Y540, V531, F527, F526, Y448, F449, W559, T456, Y460, Y461, E467 and back to K545 (Figure 3c–d). With 1.0 basic H-bonds energetically equivalent to this least-stable E467-K545 salt bridge, the calculated T_m_ for its heat unfolding was about 30 °C (Table 1), which was lower than the calculated T_m_ of 40 °C of the oxidized closed mTRPV3 channel in cNW11 nanodiscs at 42 °C (2). Therefore, oxidized hTRPV3 in cNW30 nanodiscs can remain closed until above 30 °C.

### THCV binding stabilizes oxidized hTRPV3 in the open state at 4 °C

**Figure 4:**
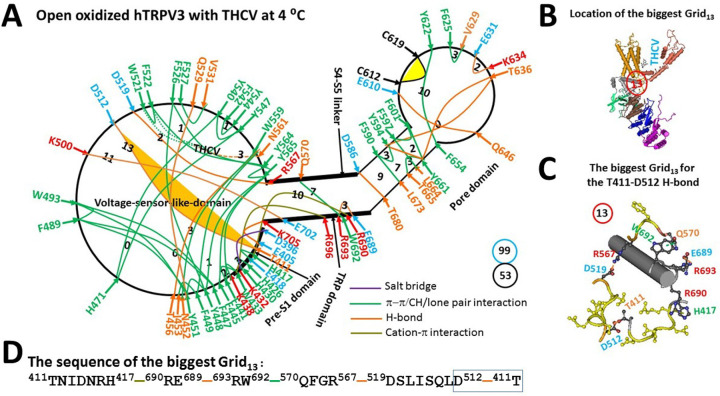
The grid-like noncovalently interacting mesh network along the PC-dependent minimal gating pathway of the oxidized hTRPV3 channel with THCV bound in an open state at 4 °C. (**A)** The thermoring structure based on cryo-EM data of a single subunit of the oxidized and open hTRPV3 channel in cNW30 nanodiscs at 4 °C (PDB ID, 8V6L). The pore domain, the S4-S5 linker, the TRP domain, and the pre-S1 domain are indicated by black arrows except the VSLD. Salt bridges, p interactions, and H-bonds between paired amino acid side chains along the PC-dependent minimal gating pathway from D396 to K705 are marked in purple, green, and orange, respectively. The specific grid sizes needed to control the least-stable noncovalent interactions in the grids are labeled with black numbers. The T411-D512 H-bond in the biggest Grid_13_ is highlighted. The total grid sizes and the total grid size-controlled noncovalent interactions along the PC-dependent minimal gating pathway are shown in the cyan and black circles, respectively. (**B)** The location of the biggest Grid_13_. (**C)** The structure of the biggest Grid_13_ with a 13-residue size to control the T411-D512 H-bond at the pre-S1/VSLD interface. (**D)** The sequence of the biggest Grid_13_ to control the T411-D512 H-bond highlighted in the blue box.

Closed oxidized hTRPV3 in cNW30 nanodiscs was unstable when the PC lipid only attached to W521. When THCV outcompeted the PC lipid from the active vanilloid site, it was wrapped by W521, F522 and N561 via two p interactions and an H-bond. In addition, in agreement with the loss of the D9-R16 salt bridge of the Trp-cage miniprotein at low temperature (7), the E467-K545 salt bridge in the biggest Grid_17’_ was disrupted (Figure 4a). In that case, as the total numbers of both grid sizes and noncovalent interactions increased from 83 and 44 to 99 and 53, respectively (Figures 3a, 4a), the systematic thermal instability (T_i_) was 1.89, similar to 1.87 of closed oxidized hTRPV3 (Table 1).

Although the stimulatory D519-R567 H-bond in the open state of oxidized mTRPV3 in cNW11 nanodiscs at 42 °C was also present in the open oxidized hTRPV3 in cNW30 nanodiscs at 4 °C (2), the presence of new K500-E702 and Y451-Q529 H-bonds allowed the new least-stable D512-T411 H-bond to be controlled by the biggest Grid_13_ for channel opening in cNW30 nanodiscs at 4 °C (Figure 4a–b). It featured a 13-residue thermoring from T411 to H417, R690, E689, R693, W692, Q570, R567, D519, D512, and back to T411 (Figure 4c–d). With a basic H-bond energetically equialent to the least-stable T611-D512 bridge at the protein-water interface (Figure 4b), the calculated T_m_ to unfold it was about 38 °C (Table 1). Thus, the replacement of PC with THCV at the active vanilloid site stabilized the open state of oxidized hTRPV3 in cNW30 nanodiscs.

### Inactivation further stabilizes oxidized hTRPV3 at 4 °C

**Figure 5:**
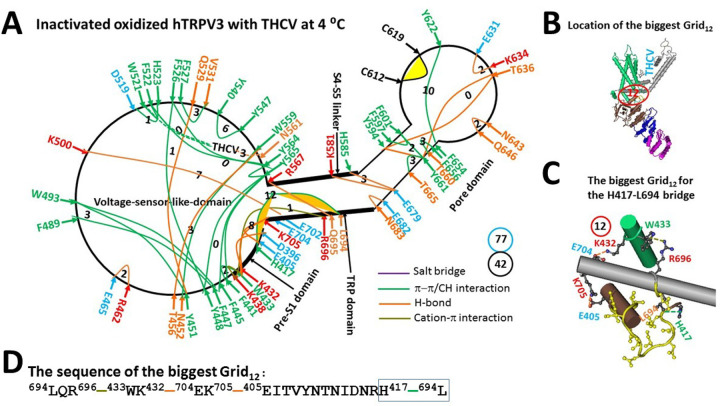
The grid-like noncovalently interacting mesh network along the PC-dependent minimal gating pathway of the oxidized hTRPV3 channel with THCV bound in an inactivated state at 4 °C. (**A)** The thermoring structure based on cryo-EM data of a single subunit of the oxidized and inactivated hTRPV3 channel in cNW30 nanodiscs at 4 °C (PDB ID, 8V6M). The pore domain, the S4-S5 linker, the TRP domain, and the pre-S1 domain are indicated by black arrows except the VSLD. Salt bridges, p interactions, and H-bonds between paired amino acid side chains along the PC-dependent minimal gating pathway from D396 to K705 are marked in purple, green, and orange, respectively. The specific grid sizes needed to control the least-stable noncovalent interactions in the grids are labeled with black numbers. The H417-L694 p interaction in the biggest Grid_12_ is highlighted. The total grid sizes and the total grid size-controlled noncovalent interactions along the PC-dependent minimal gating pathway are shown in the cyan and black circles, respectively. (**B)** The location of the biggest Grid_12_ (**C)** The structure of the biggest Grid_12_ with a 12-residue size to control the H417-L694 p interaction at the pre-S1/TRP interface. (**D)** The sequence of the biggest Grid_13_ to control the H417-L694 p interaction highlighted in the blue box.

In the inactivated state, the least-stable D512-T411 H-bond at the protein/water interface was disrupted, possibly due to the competitive H-bond between the water molecule and the side chain of the negatively-charged residue D512. Meanwhile, the stimulatory D519-R567 H-bond at the S4-S5 linker/VSLD interface was also broken but the inhibitory H417-L694 p interaction at the pre-S1/TRP interface was present as the least-stable in the biggest Grid_12_ (Figure 5a–b). It had a 12-residue thermoring from H417 to E405, K705, E704, K432, W433, R696, L694 and back to H417 (Figure 5c–d). For a basic H-bond to be energetically equivalent to the least-stable H417-L694 bridge, the calculated T_m_ to unfold it was about 40 °C, slightly higher than the 38 °C of the open state (Table 1).

Of special note, the non-native structure was present in the inactivated state due to the disconnection of the highly conserved D586-T680 and F590-L673 bridges in the normal closed and open states of oxidized mTRPV3 in cNW30 nanodiscs at 42 °C (Figure 5a) (2). After reducing the total number of grid sizes and noncovalent interactions from 96 and 53 to 77 and 42, respectively, the systematic thermal instability (T_i_) also decreased from 1.87 to 1.83 (Table 1). Thus, both the increased T_m_ and decreased T_i_ indicated that the inactivated state was more stable than the open state in the presence of THCV.

## Discussion

The thermosensitive TRPV1–4 channels are well-known for their specific activation threshold and high temperature sensitivity (Q_10_). While the former can be explained by the melting temperature threshold of the least-stable noncovalent interaction along the lipid-dependent minimal gating pathway from the pre-S1 domain to the TRP domain (2, 6), the energy origin of the high Q_10_ is still not well understood. Although a single Gibbs-Helmholtz equation has linked both cold and hot sensation of TRPV1–4 channels to produce a significant ΔC_p_ (3), cold activation has not been confirmed for the native construct without any mutation. Since the heat capacity difference (ΔC_p_) underlies heat sensing of TRPV3 (2), cold activation should be detected. In this study, asymmetric cold and heat unfolding pathways of proteins were used to examine this model (4). Once the primary gating state-dependent quaternary structures of oxidized hTRPV3 were identified at low and high temperatures, thermoring structural analyses demonstrated that these gating states had distinct thermostabilities. Therefore, the resultant temperature-dependent gating pathways of oxidized TRPV3 confirmed this heat capacity model.

### Gating state-dependent quaternary structures at low and high temperatures

**Figure 6:**
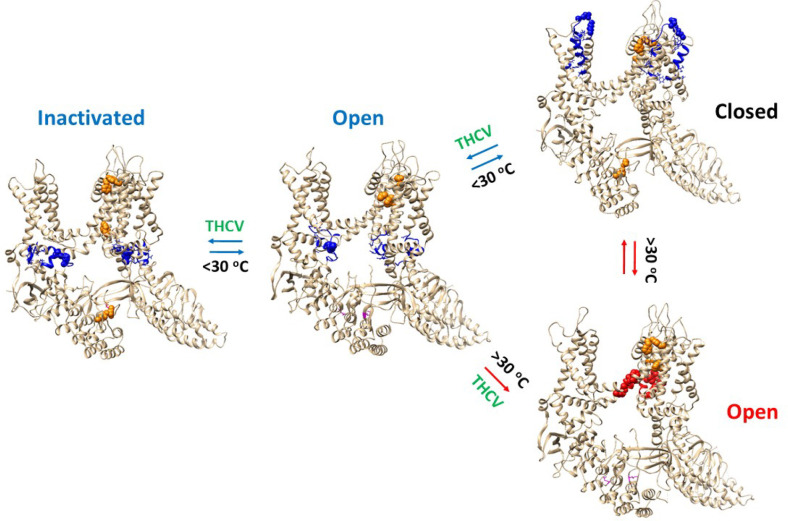
Temperature-dependent gating pathways of oxidized TRPV3. The homo-tetrameric cryo-EM structures of oxidized hTRPV3 in the PC-bound closed state, the THCV bound open and inactivated states at 4 °C (PDB ID, 8V6K, 8V6L and 8V6M, respectively) were used for the model. The homo-tetrameric cryo-EM structures of the oxidized mTRPV3 channel in the open state at 42 °C (PDB ID, 7MIO) were used as controls. The biggest thermorings Grid_17’_, Grid_13_ and Grid_12_ in the closed, open and inactivated state below 30 °C are colored blue, respectively. In contrast, the biggest thermoring Grid_9_ in the open state above 30 °C is colored red. All the least-stable noncovalent interactions controlled by those biggest thermorings are indicated by space fill. The intersubunit interactions are colored orange and indicated by space fill.

Although homotetrameric oxidized hTRPV3 channels have conserved secondary structures, their quaternary structures were different in various gating states (Figs. 1–2). Given that oxidized hTRPV3 in cNW30 nanodiscs at 4°C shared the same R416-D519 and Y594-T636-Y661-Y594 H-bonds with oxidized mTRPV3 in cNW11 nanodiscs at 42°C in the closed state with PC bound at the active vanilloid site (Fig. 3a) (2), it is reasonable that they had the same swapping Y460-F626”’ and I637-F666”’ π interactions (Figs. 1–2, 6). In addition, both closed states had the same swapping K169-D752 salt bridge (Fig. 6), in agreement with previous reports (19–20). Once THCV outcompeted the PC lipid from the active vanilloid site, the open state of oxidized hTRPV3 at 4°C had the same stimulatory D519-R567 H-bond as shown in the heat-opened oxidized mTRPV3 at 42°C (Fig. 4a) (2). However, THCV-opened oxidized hTRPV3 at 4°C had the T411-D512 H-bond in the first biggest Grid_13_ and the K500-E702 H-bond in the second biggest Grid_11_ (Fig. 4a). Thus, it is reasonable that both open states at 4°C and 42°C had the same swapping W559-L599”’ π interactions but different swapping interactions: the Y460-F625”’ π bridge at 42°C and the S459-S607”’ H-bond at 4°C (Figs. 1–2, 6). Meanwhile, the swapping K169-D752’ salt bridge was broken in both open states (Fig. 6). In the THCV-induced inactivated state at 4°C, the same swapping K169-D752’ H-bond and Y460-F625”’ π interaction in closed oxidized hTRPV3 were also observed. However, the swapping N671-Y575’ π interaction was different than the swapping I631-F666”’ π interaction (Figs. 1–2, 6). This difference may be due to the presence of the H417-L694 π interaction in the biggest Grid_12_ and the absence of the D586-T680 and F590-L673 bridges in the inactivated state (Figs. 3a, 5a). Together, gating state-dependent quaternary structures of oxidized TRPV3 at low and high temperatures may result from distinct tertiary thermoring structures in various gating states.

### Gating state-dependent thermostability

Further thermodynamic analyses showed that each gating state of oxidized hTRPV3 in cNW30 nanodiscs at 4°C and 42°C had different thermostability. Even in the presence of the PC lipid at the active vanilloid site, the closed state had a calculated melting temperature threshold (T_m_) of 30°C (Table 1). Upon a ligand exchange of PC by THCV at the vanilloid site, the T_m_ values of the open and inactivated states rose to 38°C and 40°C, respectively (Table 1). In contrast, the closed and open states of oxidized mTRPV3 in cNW11 nanodiscs have T_m_ values of 40 and 61°C, respectively (2). Notably, although the three gating states of oxidized hTRPV3 in cNW30 nanodiscs at 4°C had similar systematic thermal instability (Ti) around 1.86 (Table 1), the closed and open states of oxidized mTRPV3 in cNW11 nanodiscs at 42°C have a lower T_i_ of 1.20 (2). In this regard, these gating-state-dependent thermal stabilities at low and high temperatures may result in temperature-dependent gating pathways.

### Temperature-dependent gating pathways of oxidized hTRPV3 upon THCV binding

Based on the thermoring analysis in this study, it was found that oxidized hTRPV3 can remain closed in cNW30 nanodiscs when the temperature is below 30°C. If the same heat-evoked open state of oxidized mTRPV3 in cNW11 nanodiscs is applied to oxidized hTRPV3 in cNW30 nanodiscs, the release of PC from the active vanilloid site due to heat could also open the channel at least above 30°C (Fig. 6). In agreement with this proposal, oxidized mTRPV3 can be activated above 30°C after a prolonged heat exposure (10). Consequently, if a ligand such as THCV displaces the PC lipid from the active vanilloid site, oxidized hTRPV3 could potentially be activated by both heat and cold stimuli through different gating pathways.

Given that oxidized hTRPV3 had a similar T_i_ of 1.86 in the PC-bound closed, THCV-opened and inactivated states at 4°C (Table 1), these gating states may coexist at low temperature as recently proposed (11). However, their T_m_ values were 30, 38, and 40°C, respectively (Table 1). Therefore, the balance may shift from the close state to the open one and then the inactivated one below 30°C (Fig. 6).

On the other hand, oxidized mTRPV3 has a T_m_ of 61°C and a T_i_ of 1.20 (Table 1) (2). Therefore, an increase in thermostability may allow oxidized TRPV3 in cNW30 nanodiscs to be opened above 30°C without any inactivation even in the presence of THCV (Fig. 6). Taken together, the temperature-dependent gating pathways of oxidized TRPV3 upon ligand exchange at the active vanilloid site demonstrated that both cold and hot sensing abilities can be linked and explained by the single Gibbs-Helmholtz equation for a significant ΔC_p_.

In support of the above proposal, human TRP subtype A1 (hTRPA1) has been shown as an intrinsic redox state-dependent bidirectional thermosensor with a temperature-dependent gating pathway. Unlike cold activation below 20°C, its heat activation above 22°C is accompanied by inactivation or desensitization (21–22). Therefore, different structural rearrangements rather than identical conformational changes are involved in the dual but asymmetric cold and heat sensation of thermosensitive TRP channels to generate a temperature-dependent ΔC_p_ (23).

If the open state of oxidized mTRPV3 at 42°C is used for the heat-evoked channel opening of oxidized hTRPV3 above 30°C along the PC-dependent minimal gating pathway from D396 to K705, both the increase in total noncovalent interactions from 44 to 49 and the decrease in total grid sizes from 83 to 59 would increase the heat capacity. In contrast, for THCV-induced cold activation of oxidized hTRPV3, the increase in total noncovalent interactions from 44 to 53 would increase the heat capacity. However, the increase in total grid sizes from 83 to 99 would decrease the heat capacity (Table 1). Therefore, the ΔC_p_ at high temperature would be higher than at low temperature during channel activation. Together, the temperature-dependent gating pathway of oxidized TRPV3 confirms the heat capacity model. On the other hand, given that inactivation by THCV at low temperature was actually absent at 37°C, the structural and functional characterization of the drug binding to a target is necessary at human body temperature.

## Conclusions

Complex protein-lipid interactions make it challenging to understand the heat-sensing mechanism of the thermosensitive TRPV1–4 channels. Based on the Gibbs-Helmholtz equation, cooperative two-state unfolding of proteins observed at high temperatures is predicted to become fragmented unfolding in cold denaturation. Therefore, this temperature-dependent unfolding behavior can be used to examine whether hot and cold sensation of thermosensitive TRPV channels can be linked and explained by a single Gibbs-Helmholtz equation for a significant ΔC_p_. In this study, cryo-EM structures of oxidized TRPV3 with or without tetrahydrocannabivarin (THCV) bound at low and high temperatures were analyzed using a highly-sensitive thermodynamic method. Following the identification of the temperature-dependent gating pathway of oxidized TRPV3 upon a ligand exchange, this study supported the heat capacity model.

## Methods

### Data mining resources

The cryo-EM 3D structures of the closed, open and inactivated hTRPV3 channels with the C612-C619 disulfide bond in cNW30 nanodiscs at 4°C were examined to reveal cold-evoked gating transitions upon tetrahydrocannabivarin (THCN) binding (PDB ID, 8V6K, model resolution = 2.46 Å; 8V6L, model resolution = 3.68 Å, and 8V6M, model resolution = 3.63 Å, respectively) (11). In addition, the cryo-EM 3D structures of the closed and open mTRPV3 channels with the C612-C619 disulfide bond in cNW11 nanodiscs at 42°C were sampled as controls to indicate the heat-evoked channel gating transition (PDB ID, 7MIN, model resolution = 3.09 Å; 7MIO, model resolution = 3.48 Å, respectively) (10).

### Filtering noncovalent interactions

The stereo-selective or regio-selective inter-domain diagonal and intra-domain lateral noncovalent interactions along the PC-dependent minimal gating pathway of mTRPV3 or hTRPV3 from D396 to K705 were analyzed using UCSF Chimera. The interactions were filtered by the same strict and consistent standard as previously used and confirmed (2, 6, 9, 12–18). The examined noncovalent interactions included salt-bridges, lone pair/CH/cation-π interactions and H-bonds between paired amino acid side chains. Specific cutoff distances and interaction angles for the different noncovalent interactions can be found in the online Supporting Information (Table S1, S2 and S3).

### Mapping thermoring structures using graph theory

The study utilized the same protocol previously described and validated to map the systematic fluidic grid-like noncovalent interaction mesh network as the thermoring structure (2, 6, 9, 12–18). In this network, a topological grid was created with nodes representing amino acids and linked nodes representing noncovalent interactions along a single polypeptide chain. Graph theory and the Floyd–Warshall algorithm (24) were employed to determine the grid size as the shortest round path length to control the least-stable noncovalent interaction within the grid. The grid size also indicated the minimal number of side chains of free or silent amino acids or atoms that did not participate in any noncovalent interaction within the grid. Uncommon grid sizes were denoted in black numbers on the network map alongside the Grid_s_ with an s-residue size. The total numbers of noncovalent interactions (*N*) and total grid sizes (S) along the PC-dependent minimal gating pathway of hTRPV3 or mTRPV3 from D396 to K705 were calculated and displayed in black and cyan circles, respectively, next to the mesh network map for the calculation of systematic thermal instability based on the equation T_i_ = S/N, as previously examined (2, 6, 9, 12–18).

### Calculation of the melting temperature threshold for heat unfolding

The melting temperature threshold ($T_{m}$) for the heat unfolding of a given grid was calculated using the equation previously examined (2, 6, 9, 12–18):

$$T_{m}\left({}^{\circ}C\right)=34+(n-2)\times 10+(20-s)\times 2\;(1)$$

where, n represents the total number of basic H-bonds (~ 1 kcal/mol for each) energetically equivalent to the least-stable noncovalent interaction controlled by the given grid, and s is the grid size to control the least-stable noncovalent interaction of the given grid. Thus, the grid’s heat capacity will increase with a decrease in the grid size or an increase in equivalent basic H-bonds.

### Calculation of the melting temperature threshold for heat unfolding

The melting temperature threshold ($T_{m}$) for the heat unfolding of a given grid was calculated using the equation previously examined (2, 6, 9, 12–18):

$$T_{m}\left({}^{\circ}C\right)=34+(n-2)\times 10+(20-s)\times 2\;(1)$$

where, n represents the total number of basic H-bonds (~ 1 kcal/mol for each) energetically equivalent to the least-stable noncovalent interaction controlled by the given grid, and s is the grid size to control the least-stable noncovalent interaction of the given grid. Thus, the grid’s heat capacity will increase with a decrease in the grid size or an increase in equivalent basic H-bonds.

## Figures and Tables

**Table 1.** Comparison of local heat- and cold-induced thermoring structural changes of TRPV3 along the PC-dependent minimal gating pathway from D396 to K705. The comparative parameters are highlighted in bold.

**PDB ID**	7MIN	7MIO	8V6K	8V6L	8V6M
**Construct**	mTRPV3		hTRPV3		
**Lipid at the active vanilloid site**	PC	free	PC	THCV	
**Redox state**	Oxidized				
**Lipid environment**	cNW11		cNW30		
**Sampling temperature, °C**	42		4		
**Gating state**	Closed	Open	Closed	Open	Inactivated
**# of the biggest Grid_s_**	Grid_17_	Grid_9_	Grid_17_’	Grid_13_	Grid_12_
**grid size (s)**	17	9	17	13	12
**# of energetically equivalent basic H-bonds (n) controlled by Grid**_**s**_	2.0	2.5	1.0	1.0	1.0
**Total non-covalent interactions (N)**	53	49	44	53	42
**Total grid sizes (S), a.a**	64	59	83	99	77
**Calculated T_m_ °C**	**40**	**61**	**30**	**38**	**40**
**Systemic thermal instability (T_i_)**	**1.21**	**1.20**	**1.89**	**1.87**	**1.83**
**Refs for T_m_ and T_i_**	(2)	(2)			

## Data Availability

All data generated or analysed during this study are included in this published article and Supporting Information.

## Abbreviations

cryo-EM: cryogenic electron microscopy
DC_p_: heat capacity difference
PD: pore domain
Q_10_: functional thermosensitivity
P_o_: open probability
PI: phosphatidylinositol
PC: phosphatidylcholine
PD: pore domain
T_i_: systematic thermal instability
T_m_: melting temperature threshold
THCV: tetrahydrocannabivarin
TRP: transient receptor potential
TRPV: TRP vanilloid
TRPVi: (i=1, 2, 3, 4)
hTRPA1: human TRP subtype A1
hTRPV3: human TRPV3
mTRPV3: mouse TRPV3
rTRPV1: rat TRPV1
VSLD: voltage-sensor-like domain
WT: wild-type
